# Medical students' ratings of the relevance and actual implementation of interprofessional education and preferences for teaching formats: comparison by gender and prior education

**DOI:** 10.3205/zma001306

**Published:** 2020-03-16

**Authors:** Ronja Behrend, Anja Czeskleba, Torsten Rollinger, Mandy Petzold, Yadira Roa Romero, Raphael Raspe, Asja Maaz, Harm Peters

**Affiliations:** 1 Charité – Universitätsmedizin Berlin, Prodekanat für Studium und Lehre, Dieter Scheffner Fachzentrum für medizinische Hochschullehre und Ausbildungsforschung, Berlin, Germany; 2 Charité – Universitätsmedizin Berlin, Prodekanat für Studium und Lehre, Arbeitsbereich Qualitätssicherung, Berlin, Germany; 3 Charité – Universitätsmedizin Berlin, Fachschaftsinitiative Medizin, Berlin, Germany; 4 Charité – Universitätsmedizin Berlin, Prodekanat für Studium und Lehre, Team Projektsteuerung B.A. Pflege, Berlin, Germany

**Keywords:** interprofessional education, gender, diversity, medical students

## Abstract

**Objectives: **Interprofessional education is becoming increasingly important for collaboration in patient care. In the national context, there are few empirical studies on the assessment of medical students as important stakeholders in their education.

**Method: **Students (N=2,974) participated in a semester-wide online evaluation of the modular curriculum of medicine at the Charité Berlin. Socio-demographic data (including gender, completion of prior education/studies), assessments of the relevance and extent of interprofessional collaboration and preferences for interprofessional education in various teaching formats were collected.

**Results: **In total, data from 1,019 students were included in the evaluation. The relevance of interprofessional collaboration was considered high by medical students. Female students rated the relevance higher than male students. The completion of pre-education (vocational training or study) had no additional influence. The actual implementation of interprofessional education was rated equally low by female and male students. Medical students rated patient-centred, interactive small group formats as particularly suitable for interprofessional education. There were no gender differences, but the effect was more pronounced among students with vocational training.

**Conclusion:** The assessments of female and male students show a large difference between the perceived relevance and the actual implementation of interprofessional collaboration in the modular curriculum of medicine. This study provides an empirical basis for the actual implementation of interprofessional collaboration and students’ views on suitable teaching formats for interprofessional education.

## Zusammenfassung

**Zielsetzung: **Der interprofessionellen Ausbildung wird eine zunehmende Bedeutung für die Zusammenarbeit in der Patient*innenversorgung zugeschrieben. Im nationalen Kontext gibt es nur wenig empirische Untersuchungen zur Einschätzung von Medizinstudierenden als wichtige Stakeholder für ihre Ausbildung.

**Methodik:** In einer semesterübergreifenden Studiengangsevaluation des Modellstudiengangs Medizin der Charité wurden Studierende (N=2.974) online-basiert befragt. Es wurden soziodemografischen Daten (u. a. Geschlecht, Abschluss einer Ausbildung/eines Studiums) und Einschätzungen zu Relevanz und Ausmaß der Vermittlung interprofessioneller Zusammenarbeit sowie der Wunsch nach interprofessioneller Lehre in verschiedenen Lehrformaten erhoben.

**Ergebnisse: **Insgesamt gingen Daten von 1.019 Studierenden in die Auswertung ein. Die Relevanz interprofessioneller Zusammenarbeit wurde von den Medizinstudierenden als hoch eingeschätzt. Studentinnen bewerteten die Relevanz höher als Studenten, eine abgeschlossene Vorausbildung (Berufsausbildung oder Studium) hatte keinen zusätzlichen Einfluss. Der Vermittlungsumfang interprofessioneller Ausbildung wurde von weiblichen und männlichen Studierenden gleich niedrig einschätzt. Medizinstudierende schätzen patient*innenzentrierte, interaktive Kleingruppenformate als besonders geeignet für interprofessionelle Lehre ein. Hier zeigten sich keine Geschlechterunterschiede, der Effekt war ausgeprägter bei Studierenden mit beruflicher Vorbildung.

**Schlussfolgerung: **Die Einschätzung der weiblichen und männlichen Studierenden zeigt eine große Differenz zwischen eingeschätzter Relevanz und tatsächlicher Vermittlung von interprofessioneller Zusammenarbeit im Medizinstudium auf. Diese Studie liefert eine empirische Basis für eine Einschätzung des Ausmaßes und der von den Studierenden gewünschten Umsetzung in verschiedenen Lehrformaten.

## 1. Introduction

Interprofessional collaboration in health professions is playing an increasing role in the needs-based care of patients. In the field of medicine, the key role of interprofessional education in preparing for interprofessional practice has been recognized [1], [2], [3]. Although various interprofessional courses have been successfully designed and piloted for the German context in recent years [4], a number of fundamental, content-related and structural questions regarding the implementation of interprofessional education are unanswered for this context [5], [6], [7]. Medical students themselves constitute a relevant interest group for the process of further development of existing curricula, but there are hardly any empirical studies on student perspectives of interprofessional education. Therefore, in this article, ratings of the perceived relevance of interprofessional education and its actual implementation and views on future interprofessional education curriculum design of a cohort of female and male medical students and students with and without prior education will be reported.

A core element of interprofessional education is the principle of “learning with, from and about each other” [8]. The concepts of interprofessionalism and diversity have much in common. Their commonalities include their emphases on understanding, accepting and respecting the differences between individuals in terms of profession, gender and age, as well as economic and social status [9]. Cultural differences are a central dimension of diversity. Similarly, interprofessional cooperation can be understood as a combination of different professional cultures [10], [11]. Both concepts aim to go beyond working side by side. Differences should instead be actively used to promote successful coexistence. Regarding the social context, both require a safe, positive and beneficial environment.

Interprofessional education is a relatively recent and under-implemented topic in Germany, especially in medical education. “Operation Team”, an initiative of the Robert Bosch Stiftung, funded a number of pilot projects to develop and implement interprofessional education [4]. These pilot projects explored the implementation of interprofessional education with different content, teaching formats and faculties, mostly in small groups and cohorts, and often as extracurricular events. As part of the programme, the authors’ faculty was able to implement two interprofessional courses as part of the mandatory curriculum. Some students now have the opportunity to learn about interprofessionalism in interprofessional groups and with interprofessional lecturers [12], [13]. Furthermore, six different interprofessional tutorials can be selected and credited by the students as part of the tutorial offer [14]. Beside those pilot projects, interprofessional education is offered sparsely in the modular curriculum at Charité. 

For the development or further development of medical curricula, there has been good national and international experience in actively involving students in curriculum development. Students know the “curriculum in action”; they represent the products of their study programmes and are voting members of the academic community [15], [16], [17], [18], [19]. A key domain for student participation is curriculum design and improvement. These experiences have been described in particular for monoprofessional medical curriculum development.

Regarding the further interprofessional development of existing study programmes, there has been little published data on the students' perspectives in national contexts. In a qualitative analysis of student participation, we were able to show that the active participation of students from different professions had a positive, complementary effect on the development and design of interprofessional courses [20]. In a further work, we collected students’ assessments of the implementation of the modular curriculum of medicine (MCM) at the Charité – Universitätsmedizin Berlin (Charité) [21]. This across-semester, cross-sectional survey on 14 overall educational goals showed that interprofessional collaboration was considered relevant by 93% of medical students, but only 28% believed that interprofessional collaboration was integrated well in the medical curriculum [21]. The gap between relevance and actual implementation was greater in the “interprofessional collaboration” domain than in any of the other 14 areas. There are indications in the international literature that gender influences the subjective rating and the benefit of interprofessional education [22], [23].

Furthermore, for interprofessional education, small group formats and formats with practical and patient relevance are described as suitable [24], [25]. To gather empirical information on student assessments in a national context, the data set from the aforementioned cross-sectional study at the Charité was further analysed in light of these perspectives.

The first aim of this work is to examine how students of the MCM of the Charité rate the relevance of interprofessional collaboration for their future work as physicians and the actual implementation of interprofessional collaboration in the MCM. The research questions (FFs) are examined to determine whether there are differences in the assessments between female and male medical students (FF1.1) and between students who have or have not already completed a vocational training/studies (FF1.2). Second, the work examines the currently existing teaching formats in which students would wish to integrate interprofessional education (FF2). Additionally, for this research question, the results are examined and reported by gender (FF2.1) and the completion of vocational training/studies (FF2.2).

## 2. Method

### Setting

In the period from 2010-2016, the Charité developed and implemented an integrated, outcome-oriented and competence-based medical study programme (MCM) [26]. The development took place through a faculty-wide, standardized and transparent development process in which students participated actively. The programme comprises 40 modules over 10 semesters, followed by the practical year. Approximately 300 medical students are enrolled every semester. The MCM features longitudinal teaching formats, such as communication, interaction and teamwork (CIT) and problem-based learning (PBL). Table 1 (Tab. 1) gives an overview of the different teaching formats in the MCM. As described in the introduction, interprofessional education takes place only occasionally.

#### Data collection and sample

To evaluate the MCM, all medical students who had completed 1-10 semesters were invited to participate in an across-semester student evaluation of the study programme (N=2,974). The evaluation was administered as an online survey from November 2017 to January 2018 with EvaSys evaluation software (Electric Paper Evaluationssysteme GmbH, Lüneburg, Germany). In total, four reminder emails were sent at weekly intervals after an invitation email to participate in the survey was sent. In addition, posters were placed on campus, and calls for participation were posted in semester groups on Facebook. Participation in the survey was voluntary, and pseudonyms were used for participants. The study received approval from the Charité office for data protection.

#### Questionnaire

Together with the quality assurance department and the Dieter Scheffner Center for Medical Education, the students' medical advisory board developed a comprehensive questionnaire that includes free text commentary, single and multiple choice questions and scale questions (5-point Likert scale). Various socio-demographic data were collected, including gender and level of completion of prior education/studies before starting medical studies. The questionnaire included questions on the integration of basic science teaching, clinical teaching and patient-based teaching into the study programme; students’ satisfaction with longitudinal teaching formats (e.g., CIT and PBL); the relevance and actual implementation of the outcomes defined for the MCM (supplemented with gender sensitive and culturally sensitive patient interaction and interprofessional education); and students’ readiness for interprofessional learning. Attachment 1 contains the questionnaire items evaluated for this publication.

#### Data analysis

The distributions of the relevance and extent of implementation and preferences for interprofessional teaching formats were calculated with descriptive statistics. To examine whether female and male students (F1.1) and students who had and had not previously completed vocational training/studies (FF1.2) differed in their ratings of the relevance and extent of implementation of interprofessional collaboration in the MCM, two-sample t-tests were used. A two-sample t-test can be used to test hypotheses and determine whether there are differences between two groups based on their means. Based on a series of χ^2^ distribution tests, the desire for interprofessional education was examined with regard to gender (male/female; FF2.1) and prior education (completed vocational training/study vs. no vocational training/study programme started or completed) (FF2.2). To address the problem of multiple testing, the p-values were corrected using the Benjamini-Hochberg procedure [27].

Due to the exploratory nature of the study, the *p*-value information should be interpreted descriptively. Descriptive and exploratory inference statistics calculations were performed using IBM SPSS Version 24.

## 3. Results

### Returns

A total of 1,019 of the invited 2,974 MCM students participated in the survey (return rate of 34.3%). Of these participants, 653 (64.1%) reported their gender as “female”, 345 (33.9%) reported “male”, four (0.4%) reported “other”, and 17 (1.6%) did not answer the question. The “female”/“male” ratio corresponded to the current gender distribution in the study programme (64.5% female, 35.5% male) [28]. Due to the small group size, those who did not report either a female or a male gender were excluded from the evaluation.

In total, 215 students (21.1%) stated that they had already completed prior education (vocational training or studies). In our analyses, this group was compared to the group of students who had not started or completed any prior education (n=659). Those who did not specify their completion of education (n=13) or who had started vocational training/studies but had not completed it (n=132) were excluded because the data did not show how long the education/studies had lasted.

Students reported a broad range of types of completed vocational training or study programmes; there was, however, an accumulation in health education fields, such as nursing (n=65), paramedic (n=17), physical/occupational therapy (n=13) and psychology (n=16).

#### FF1: Assessment of the relevance and actual implementation of interprofessional collaboration

##### Comparison of women and men

**Figure 1:**
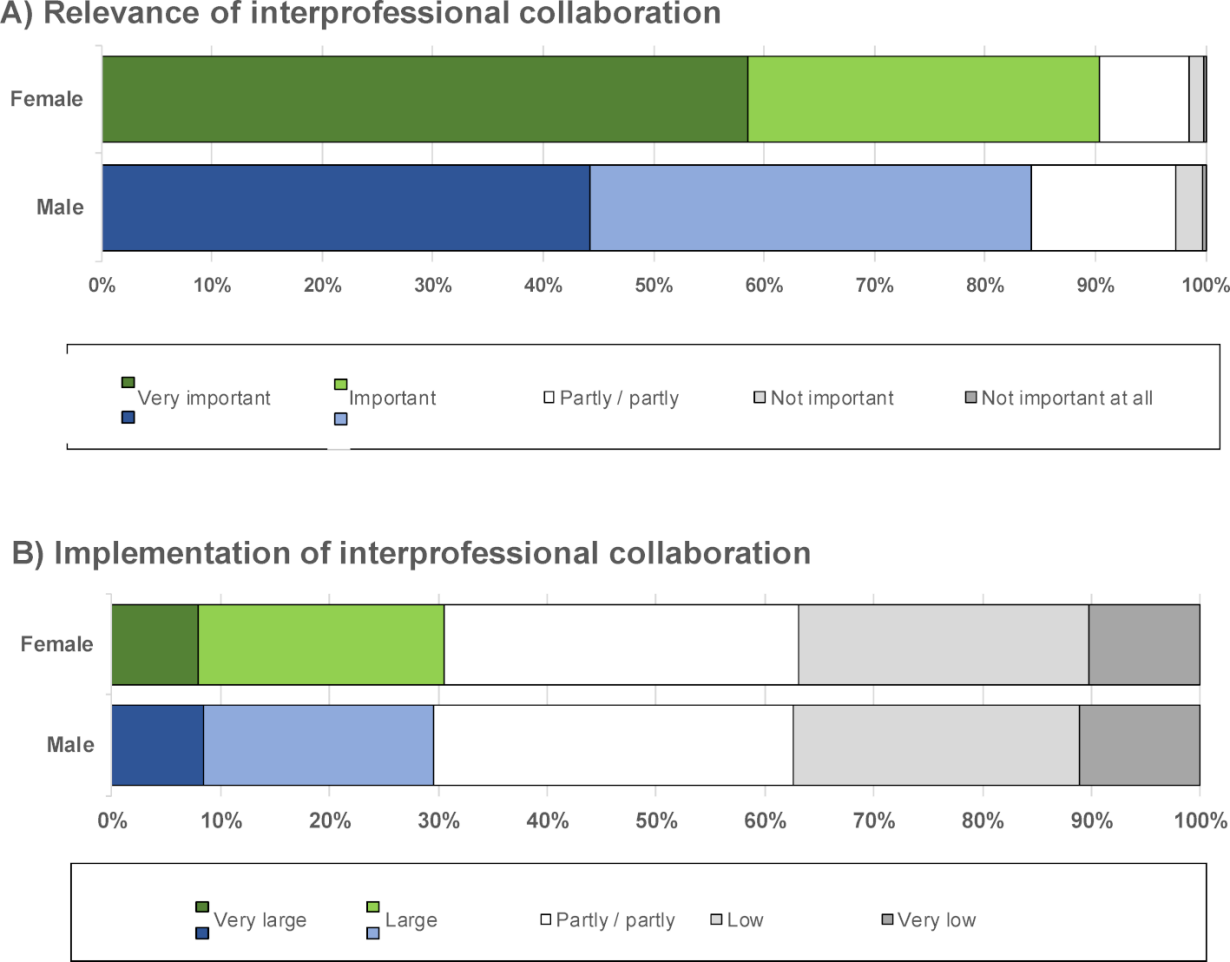
Perceived relevance (A) and actual implementation (B) of interprofessional collaboration in the MCM – Comparison of women and men (FF1.1).

As described in the introduction, the overall cohort of students in the MCM indicated their perceived relevance of interprofessional collaboration to be high [21]. A gender comparison of the same cohort revealed that women rated the relevance significantly higher than men (*t*(966)=4.25, *p*<.001, *M*_w_=4.47, *SD*_w_=.72, *M*_m_=4.25, *SD*_m_=0.80). The effect size of the difference (Cohen’s *d*=0.29) corresponded to a small effect as described by Cohen [29]. Regarding the extent of actual implementation, there were no differences between female (*M*=2.92, *SD*=1.10) and male (*M*=2.89, *SD*=1.11) students (*t*(913)=0.31, *p*=.755) (see figure 1 (Fig. 1)).

##### Comparison of students who had and had not completed vocational training/studies

In addition, students who had completed vocational training or studies were compared with those who had not. There were no differences between students with and without previous education in regard to their perceptions of relevance and actual implementation of interprofessional education (relevance: *t*(847)=0.22; *p*=.826; *M*_no_=4.40; *SD*_no_=0.76; *M*_fin_=4.41; *SD*_fin_=0.73; actual implementation:* t*(798)=0.47; *p*=.637; *M*_no_ 2.90; *SD*_no_=1.11; *M*_fin_=2.86; *SD*_fin_=1.06).

#### FF2: Desires to learn with trainees/students from other health professions in the various teaching formats

##### Comparison of women and men

**Figure 2:**
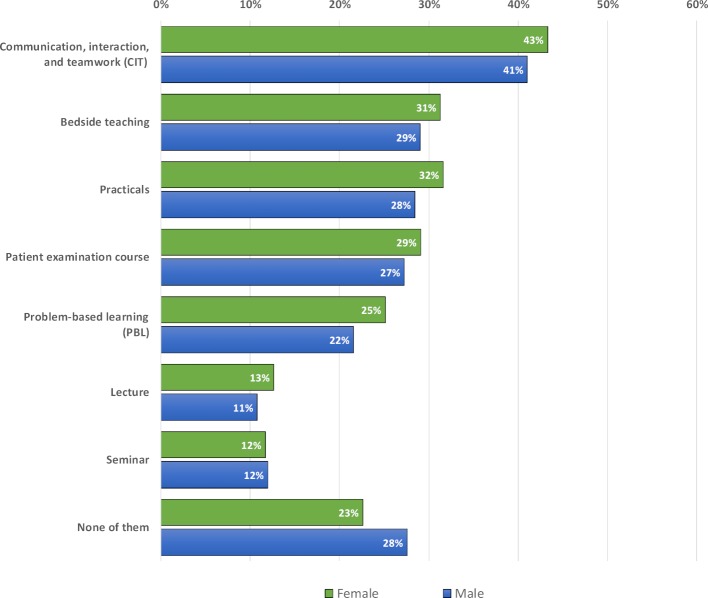
Desires to learn with trainees/students from other health professions in the various teaching formats – Comparison of women and men (FF2.1).

More than three-quarters of female students (77.4%) stated that they wanted to learn interprofessionally with trainees/students from other health professions in at least one teaching format. A total of 72.5% of male students agreed on at least one preferred teaching format (see figure 2 (Fig. 2)). Both genders preferred CIT, a longitudinal small group format for interprofessional education, followed by bedside teaching and practicals. In most teaching formats, more women than men wanted to learn with students or trainees from other health professions. Men also tended to more often desire interprofessional education in any format. None of the observed approval rates differed significantly from the expected frequencies.

##### Comparison of students who had and had not completed vocational training/studies

**Figure 3:**
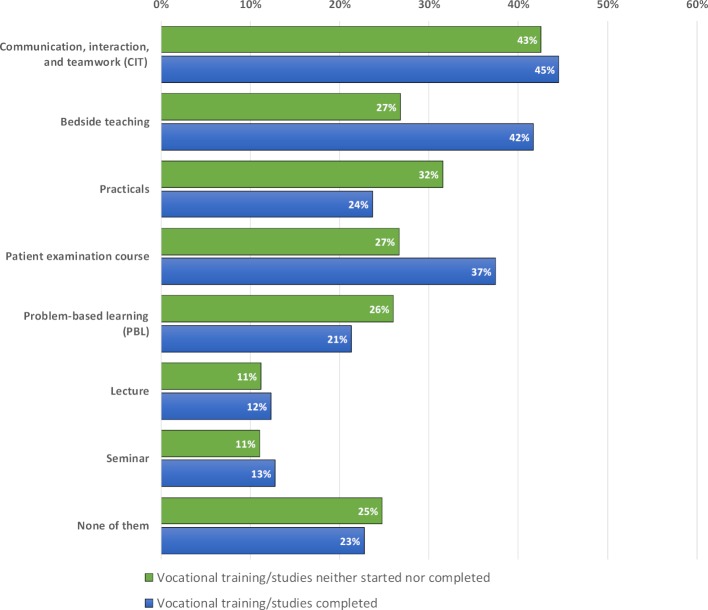
Desires to learn with trainees/students from other health professions in the various teaching formats – Comparison of students who had and had not completed vocational training/studies (FF2.2).

While 75.2% of students without vocational training or studies desired interprofessional education in at least one teaching format, 77.3% of students with vocational training or studies said they want to be taught interprofessionally in at least one teaching format (see figure 3 (Fig. 3)). Students with prior education preferred, in particular, clinical-practical teaching formats such as bedside teaching and patient examination courses with interprofessional education. The statistical analyses showed that the observed and expected frequencies differed significantly concerning the desire for interprofessional education in patient examination courses (χ^2^(1)=8.88, *p*=.012) and bedside teaching (χ^2^(1)=16.55; *p*=.001).

## 4. Discussion

The aim of this study was to determine the perceived relevance and actual implementation of interprofessional collaboration as well as potential curricular implementation from the point of view of female and male medical students. Overall, the students clearly rate the relevance of interprofessional collaboration in terms of teaching content significantly higher than its actual implementation in the medical curriculum. This effect is reinforced by differentiating the relevance of interprofessional collaboration by gender. For example, women perceive the relevance of interprofessional collaboration to be higher than men. The perceived extent of actual implementation, on the other hand, is equally low for women and men. As this study shows, there is a need from a student's point of view – irrespective of gender – for interprofessional collaboration as teaching content. This identified need from the students’ perspective coincides with and supports the demand of education and health experts for the qualification of medical students in interprofessional collaboration [2], [5].

Female and male medical students both consider practice-oriented, small group formats, which offer opportunity for interactive discussion to be suitable for interprofessional education. This finding is in line with international literature, which recommends formats that have practical relevance and allow for the direct exchange of participants, as well as learning with, from and about one another [24], [25]. Men more often tend to prefer interprofessional education in any teaching format. Lindh Falk and colleagues [22] as well as Wilhelmsson and colleagues [23] reported similar findings, observing that male students had less favourable views towards interprofessional education.

Students who have already completed vocational training or studies more often prefer interprofessional education in bedside teaching and patient examination course formats, which have strong patient and practical relevance, than students without prior education. This finding could be explained by the fact that students differently assess the importance of interprofessional collaboration for patient care because of their previous professional experience in health care.

A special feature of this study is that it uses quantitative methods and a large number of female and male students to show how large the gap between the need for interprofessional education and its actual implementation in the medical curriculum truly is. The increased integration of interprofessional education is one of the key elements in the concept recently approved by the Berlin Senate for the further development of the MCM. To further develop interprofessional education, an interprofessional network, “Charité Network for Interprofessional Education”, was launched in 2018. One goal is to implement a longitudinal, interprofessional curriculum for the MCM in the coming years. For this purpose, overarching educational goals (outcomes) for all health professions are being developed with the active participation of students. These outcomes are meant to be the basis for the interprofessional curriculum. As it is tradition at the Charité, students are included actively in all stages of development as experts of their own curriculum.

The present study has several limitations. Medical students from only one medical programme at one medical school were interviewed. The transferability of the conclusions to students in traditional study programmes or from other faculties or other professions is therefore limited. The influence of prior education was dichotomized (completed vocational training/studies vs. no preliminary education). Started vocational training/studies could also have an impact on the assessment of relevance. In the following studies, further diversity characteristics, such as the cultural background, should be considered. In particular, a survey of other professions could usefully supplement students’ perspectives and show potential existing differences.

## 5. Conclusion

Female and male medical students attach great importance to interprofessional collaboration, with female students reporting even higher scores. The extent of actual implementation is considered by female and male students to be significantly lower than the relevance of interprofessional education; thus, from a student perspective, there is a great need for interprofessional education in medical studies. Students of both genders consider interactive and patient-oriented small group formats to be particularly suitable for interprofessional education. This effect is particularly evident in students with previously completed vocational training/studies. The addition of the student perspective with the results of this empirical study complements the current discussion on interprofessional curriculum development of medical programmes in Germany.

## Acknowledgements

The authors would like to thank all the students who took part in the survey as well as the student representatives who were involved in the conception of the survey. Special thanks to all students, colleagues and the whole development team of the reformed medical curriculum at the Charité.

## Competing interests

The authors declare that they have no competing interests. 

## Supplementary Material

Extract from the questionnaire

## Figures and Tables

**Table 1:**
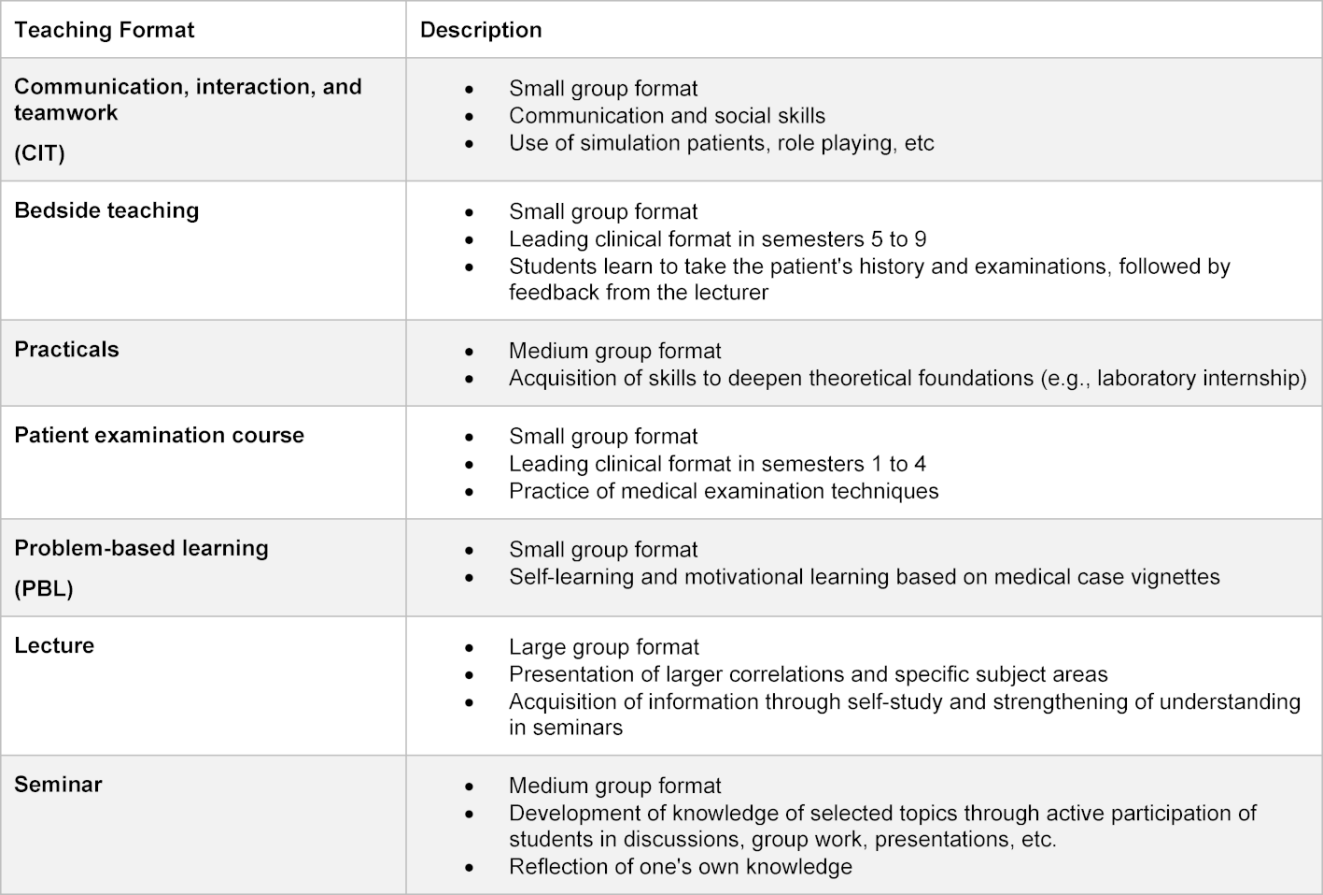
Overview of the teaching formats in the MCM at the Charité.
