# Supplementary material for: Medical students' ratings of the relevance and actual implementation of interprofessional education and preferences for teaching formats: comparison by gender and prior education
Source: GMS J Med Educ. 2020 Mar 16;37(2):Doc13. doi: 10.3205/zma001306 (PMC7171353; doi:10.3205/zma001306)
Supplement: Extract from the questionnaire [file JME-37-13-s-001.pdf]

Attachment 1: Extract from the questionaire

Which gender are you?
☐ female ☐ male ☐ another
☐ no comment

Have you already completed a previous vocational training/studies?
☐ study programme completed ☐ study programme started, but not completed ☐ vocational training completed
☐ vocational training started, but not completed ☐ no ☐ no comment

If yes, which previous vocational training/studies have you completed?

[ ]

Interprofessional education

In which teaching formats would you prefere for interprofessional education with studentsof other health professions (e.g. Nursing, Physical therapy, speech therapy)?

☐ CIT ☐ PBL ☐ Patient examination course
☐ Bedside teaching ☐ Seminar ☐ Lecture
☐ Internships ☐ Non of them

In which teaching formats would you like to be trained by teachers of other health professions (e.g. nursing, physical therapy, speech therapy)?

☐ CIT ☐ PBL ☐ Patient examination course
☐ Bedside teaching ☐ Seminar ☐ Lecture
☐ Internships ☐ Non of them

Competencies and implementation

In the following you see competency domains of professional activities. Please rate the relevance and the actual implementation in the MCM.

Interprofessional Collaboration	very important ☐	☐	☐	☐	☐ not important at all
Interprofessional Collaboration	very large ☐	☐	☐	☐	☐ very low

Attachment 1 to *Behrend R, Czeskleba A, Rollinger T, Petzold M, Roa Romero Y, Raspe R, Maaz A, Peters H. Medical students' ratings of the relevance and actual implementation of interprofessional education and preferences for teaching formats: comparison by gender and prior education. GMS J Med Educ. 2020;37(2):Doc13. DOI: 10.3205/zma001306, URN: urn:nbn:de:0183-zma0013068*
Online available from: https://www.egms.de/en/journals/zma/2020-37/zma001306.shtml
